# Optimizing perioperative care in liver disease: the role of TPO-receptor agonists

**DOI:** 10.1097/MS9.0000000000003892

**Published:** 2025-09-15

**Authors:** Archana Kumari, Muddassir Khalid, Amisha Kumari, Mohammed Hammad Jaber Amin

**Affiliations:** a Department of Medicine, Jinnah Sindh Medical University, Karachi, Pakistan; b Department of Medicine, Nishtar Medical University, Multan, Pakistan; c Department of Medicine, Liaquat University of Medical and Health Sciences, Jamshoro, Pakistan; d Department of Medicine, Alzaeim Alazhari University, Khartoum, Sudan

*Dear Editor,*

Thrombocytopenia is usually defined as any decrease in platelet count below the lower standard limit of 150 000/μL. Severe thrombocytopenia (platelet count < 50 000/μL) complicates the management of patients with chronic liver disease (CLD) by increasing the potential risk of bleeding for invasive procedures, which may therefore be delayed or canceled even if lifesaving^[1]^. Thrombocytopenia in liver disease results from increased splenic sequestration due to portal hypertension and subsequent hypersplenism, decreased thrombopoietin (TPO) production, and/or from toxic or virus-induced suppression of megakaryocytopoiesis^[2]^. Platelet transfusion is the fastest and gold-standard treatment for thrombocytopenia in patients receiving invasive procedures. However, clinicians tried to avoid its frequent use due to the disadvantages such as safety risks of transfusion, donor shortage, and high costs, especially in China. Splenectomy and splenic artery embolization are also effective in patients with CLD and thrombocytopenia. However, concerns remain regarding the serious complications after splenectomy and the recurrence of thrombocytopenia after splenic embolization^[3]^. Oral TPO receptor agonists (TPO-RAs) are an alternative management option that can be used before surgery to stimulate TPO and increase platelet count, thus reducing transfusion need. The effect of TPO agonists is mediated by their interaction with TPO receptors on megakaryocytes, which are the cells that produce platelets. This interaction triggers a series of biological pathways that ultimately lead to an increase in platelet production^[4]^.

In a prospective cohort study of 330 patients with CLD undergoing invasive procedures, 81 received avatrombopag and 173 had no platelet-raising therapy. The patients were selected based on their need for invasive procedures and their pre-procedure platelet counts. Before procedures, 78.8% of avatrombopag patients achieved platelet counts >50 × 10⁹/L and 90.9% avoided transfusion, compared with 23.4% and 63.9% in controls, demonstrating avatrombopag’s superior efficacy in perioperative platelet optimization and transfusion avoidance**^[5]^.** Similarly, in a double-masked, parallel-group phase 3 study conducted from July 2020 to June 2021, 66 patients were randomized into two groups: the lusutrombopag group (*n* = 44, 3 mg for 7 consecutive days, once daily) and placebo (*n* = 22). The proportion of patients with PLT ≥ 50 × 10^9^/L on or after Day 8 and within 2 days before procedure was 72.7% (32/44) in the lusutrombopag group and 18.2% (4/22) in the placebo group. This significant difference in platelet counts suggests that lusutrombopag is effective in raising platelet counts in patients with CLD, potentially reducing the need for platelet transfusions and improving patient outcomes^[3]^. In conclusion, TPO-RAs, including avatrombopag and lusutrombopag, safely raise platelet counts in CLD, enabling timely procedures and reducing transfusion needs, supporting a shift toward pharmacologic perioperative optimization.

TITAN Guidelines: This manuscript complies with TITAN Guidelines, 2025, declaring no use of artificial intelligence^[6]^.

## Data Availability

Not applicable.
